# The DNA methylation profile of non-coding RNAs improves prognosis prediction for pancreatic adenocarcinoma

**DOI:** 10.1186/s12935-019-0828-8

**Published:** 2019-04-23

**Authors:** Jie Zhang, Keqing Shi, Weiguo Huang, Wanqing Weng, Zhongjing Zhang, Yangyang Guo, Tuo Deng, Yukai Xiang, Xiaofeng Ni, Bicheng Chen, Mengtao Zhou

**Affiliations:** 1 Key Laboratory of Diagnosis and Treatment of Severe Hepato-Pancreatic Diseases of Zhejiang Province, The First Affiliated Hospital, Wenzhou Medical University, Wenzhou, 325015 Zhejiang Province People’s Republic of China; 2 Precision Medicine Center, The First Affiliated Hospital, Wenzhou Medical University, Wenzhou, 325015 Zhejiang Province People’s Republic of China

**Keywords:** DNA methylation, Pancreatic adenocarcinoma, miRNA, lncRNA, Classifier, Prognosis

## Abstract

**Background:**

Compelling lines of evidence indicate that DNA methylation of non-coding RNAs (ncRNAs) plays critical roles in various tumour progression. In addition, the differential methylation of ncRNAs can predict prognosis of patients. However, little is known about the clear relationship between DNA methylation profile of ncRNAs and the prognosis of pancreatic adenocarcinoma (PAC) patients.

**Methods:**

The data of DNA methylation, RNA-seq, miRNA-seq and clinical features of PAC patients were collected from TCGA database. The DNA methylation profile was obtained using the Infinium HumanMethylation450 BeadChip array. LASSO regression was performed to construct two methylation-based classifiers. The risk score of methylation-based classifiers was calculated for each patient, and the accuracy of the classifiers in predicting overall survival (OS) was examined by ROC curve analysis. In addition, Cox regression models were utilized to assess whether clinical variables and the classifiers were independent prognostic factors for OS. The targets of miRNA and the genes co-expressed with lncRNA were identified with DIANA microT-CDS and the Multi-Experiment Matrix (MEM), respectively. Moreover, DAVID Bioinformatics Resources were applied to analyse the functional enrichment of these targets and co-expressed genes.

**Results:**

A total of 4004 CpG sites of miRNA and 11,259 CpG sites of lncRNA were screened. Among these CpG sites, 8 CpG sites of miRNA and 7 CpG sites of lncRNA were found with regression coefficients. By multiplying the sum of methylation degrees of the selected CpGs with these coefficients, two methylation-based classifiers were constructed. The classifiers have shown good performance in predicting the survival rate of PAC patients at varying follow-up times. Interestingly, both of these two classifiers were predominant and independent factors for OS. Furthermore, functional enrichment analysis demonstrated that aberrantly methylated miRNAs and lncRNAs are related to calcium ion transmembrane transport and MAPK, Ras and calcium signalling pathways.

**Conclusion:**

In the present study, we identified two methylation-based classifiers of ncRNA associated with OS in PAC patients through a comprehensive analysis of miRNA and lncRNA profiles. We are the first group to demonstrate a relationship between the aberrant DNA methylation of ncRNAs and the prognosis of PAC, and this relationship would contribute to individualized PAC therapy.

**Electronic supplementary material:**

The online version of this article (10.1186/s12935-019-0828-8) contains supplementary material, which is available to authorized users.

## Background

Pancreatic adenocarcinoma (PAC) is one of the most prevalent type of highly lethal malignancy in the digestive system. It is the fourth most dominant cancer-associated death, with a 5-year survival rate less than 5% [1]. Ttreatment of PAC, including surgical resection, radiotherapy and chemotherapy, has been improved in the recent years [2]. However, even with these disease management options, the overall survival (OS) rates of PAC patients are still far from satisfactory. The poor effectiveness of treatment therapies results from the high aggressiveness of PAC, its resistance to chemotherapy drugs, and a lack of early diagnosis [3]. Therefore, to reduce mortality and improve the management of PAC patients, identification of prognosis biomarkers during the early stage of PAC is extremely important.

Non-coding RNAs (ncRNAs) have been widely found in complex eukaryotic organisms, and have gradually emerged as a new research focus because of their unique biological roles in tumour progression [4]. Although they cannot be translated into proteins, ncRNAs play critical roles in chromosomal modification, transcriptional interference, post-transcriptional modification and translational regulation via epigenetic modifications in human cells [5]. Emerging evidence has revealed that ncRNAs with dysregulated expression profiles are involved in the pathogenesis of multiple cancers, including colorectal cancer [6], breast cancer [7], as well as gastric cancer [8]. Furthermore, epigenetic modifications, especially DNA methylation, have been determined to play pivotal roles in the regulation of protein-encoding genes, miRNAs and lncRNAs [9]. Zhang et al. [10] demonstrated that the tumour suppressor microRNA-596 (miR-596) was silenced by hypermethylation at its relevant promoter in gastric cancer but not in normal tissues. Furthermore, the miR-596 expression of gastric cancer cell lines was reduced, accompanied by an increment of miR-596 methylation, which is consistent with previous study. miR-770 has also been reported to be significantly decreased in gastric cardia adenocarcinoma (GCA) as a result of the hypermethylation of the lncRNA coded by MEG3. Additionally, the aberrant methylated CpG sites of MEG3 and dysregulated miR-770 content were closely related to a poor prognosis for patients [11]. These studies indicated that abnormal DNA methylation could be a vital biomarker in the pathophysiology and evaluation of cancers. Hence, we hypothesize that the methylation of ncRNAs can predict the prognosis of PAC.

To date, the prognostic values assessed by the methylation of ncRNAs and the association between miRNAs and lncRNAs in PAC remain poorly elucidated. In our present study, differential methylation patterns of miRNA and lncRNA were analysed in PAC using the 485,577 sites identified by the HumanMethylation450 BeadChip, which is annotated by The Cancer Genome Atlas (TCGA). Using the Least Absolute Shrinkage and Selection Operator (LASSO) regression model, we successfully developed miRNA and lncRNA methylation-based classifiers to assess OS of patients. Functional enrichment analysis of differently methylated ncRNAs was performed to analyse the target genes and the association between miRNAs and lncRNAs.

## Materials and methods

### Patient datasets

The data of DNA methylation, RNA-seq, miRNA-seq and clinical features in PAC patients were from TCGA (https://portal.gdc.cancer.gov/). DNA methylation profiles was determined via the Infinium HumanMethylation450 BeadChip. Additionally, RNA-seq and miRNA-seq were processed on the Illumina HiSeq RNA-seq and miRNA-seq platforms, respectively. The lncRNA annotation file was obtained from GENCODE (https://www.gencodegenes.org/). According to the publication guidelines, all of the datasets used in this study were publicly available.

### CpG sites of miRNAs and lncRNAs

The extent of methylation of a CpG site is characterized using a beta-value, ranging from 0 (unmethylated) to 1 (completely methylated). We used the “minfi” package in R software for normalization of methylation beta-values. On the basis of the annotations by the TCGA, CpG sites within 2 kb upstream of the transcriptional start site (TSS) of lncRNA or miRNA were chosen from the 485,577 sites of the HumanMethylation450 BeadChip. Using the “minfi” package in R software, miRNA and lncRNA sites that were differentially methylated between PAC tissues and normal adjacent tissues were identified, and q-values < 0.05 were considered statistically significant. To calculate the different beta-values of CpG sites between PAC tissues and normal adjacent tissues, a *t* test was performed and beta-values differences greater than 0.1 were considered significant. Afterwards, correlation analysis was applied to identify these sites to determine where methylation levels were negatively correlated with the expression of miRNA or lncRNA. The results were adopted when the P-values was less than 0.05.

### Methylation-based classifiers for overall survival

The LASSO regression model [12] was used to identify the most accurate predictive CpGs. For example, if there were two different methylated CpGs sites in parallel, LASSO would automatically filter out the secondary related one and assign the selected CpGs site a value, which equals the regression coefficient in the classifier formulas. Meanwhile, the beta value in the formulas represents the methylation degree of the selected CpG site of miRNA or lncRNA. Herein, with the unique method of LASSO by the “glmnet” package in R software, we made the sum of the methylation degrees of the selected CpGs weighted by the coefficients and developed a linear model under high dimensional conditions with the least regression coefficients to maximize the prediction accuracy in PAC prognosis.

### Predictive and prognostic analysis of the methylation-based classifiers

Patient risk scores were determined based on the methylation-based classifiers, and time-dependent receiver operating characteristic (tdROC) curve analysis was applied to evaluate the predictive accuracy of classifiers for OS based on the risk score. To assess whether clinical variables and the classifiers were independent prognostic factors for OS, we utilized univariate and multivariate Cox regression models to identify significant prognostic predictors associated with OS. The prognostic evaluation of clinical variables and classifiers was also performed with tdROC analysis using the “timeROC” package of R software. The predictive or prognostic accuracy was indicated by the area under the curve (AUC) of tdROC. We used the median of the classifier risk score as a cut-off point to categorized the patients into two groups, including high-risk groups and low-risk groups. The survival estimation of patients were analysed with the Kaplan–Meier method. In addition, P-values < 0.05 were considered statistically significant.

### miRNA target genes, lncRNA co-expression genes and functional enrichment analysis

The DIANA microT-CDS tool (http://diana.imis.athena-innovation.gr/) were applied to predict the target genes of miRNA. The co-expression genes of lncRNA were identified with the Multi-Experiment Matrix (MEM) [13]. MEM has a large collections of microarray data sets. Using simultaneous statistical significance estimation, MEM applies rank aggregation to merge information from different data sets into a single global ordering. We used MEM to perform a gene expression similarity search across many data sets (https://biit.cs.ut.ee/mem/). We then performed functional enrichment analysis of the target genes and co-expression genes using DAVID Bioinformatics Resources (https://david.ncifcrf.gov/). It was considered statistically significant when false discovery rates (FDRs) of enrichment terms were less than 0.05. The major enrichment terms were visualized using the “ggplot2” package in R software.

## Results

### Characteristics of patient datasets

We studied 184 PAC patients, and collected both DNA methylation data and survival data from the TCGA. Table 1 illustrated the main clinical characteristics of the 184 PAC patients. The methylation data comprised 194 samples, including 184 PAC samples and 10 samples of normal adjacent tissues for corresponding PAC patients. The miRNA-seq data included 181 samples, and the RNA-seq data included 182 samples.

**Table 1:** Clinical characteristics of pancreatic adenocarcinoma

Clinicopathological variables	n = 184
Age (years)	
< 60	57 (31.0%)
≥ 60	127 (69.0%)
Gender	
Male	102 (55.4%)
Female	82 (44.6%)
Tumor grade	
G1 + G2	129 (70.1%)
G3 + G4	53 (28.8%)
Pathological tumor size	
T1 + T2	31 (16.8%)
T3 + T4	151 (82.1%)
Pathological stage	
I + II	172 (93.5%)
III + IV	9 (4.9%)
Recurrence	83 (45.1%)
Death	99 (53.8%)

### Differential methylation of CpG sites and methylation-based classifier

A total of 4004 CpG sites of miRNA and 11,259 CpG sites of lncRNA located within 2 kb upstream of the miRNA or lncRNA TSS (excluding the CpG sites on the X and Y chromosomes) were screened according to the HumanMethylation450 BeadChip annotations in the TCGA. In total, 511 CpG sites of miRNA and 1441 CpG sites of lncRNA with different methylation patterns between PAC and normal adjacent tissues were screened and analysed using the “minfi” package in R software; 273 CpG sites of miRNA and 809 CpG sites of lncRNA had beta-value differences greater than 0.1. Among these CpG sites, the methylation levels of 86 CpG sites of miRNA and 348 CpG sites of lncRNA were negatively related to the expression levels of miRNA or lncRNA.

**Fig. 1:**
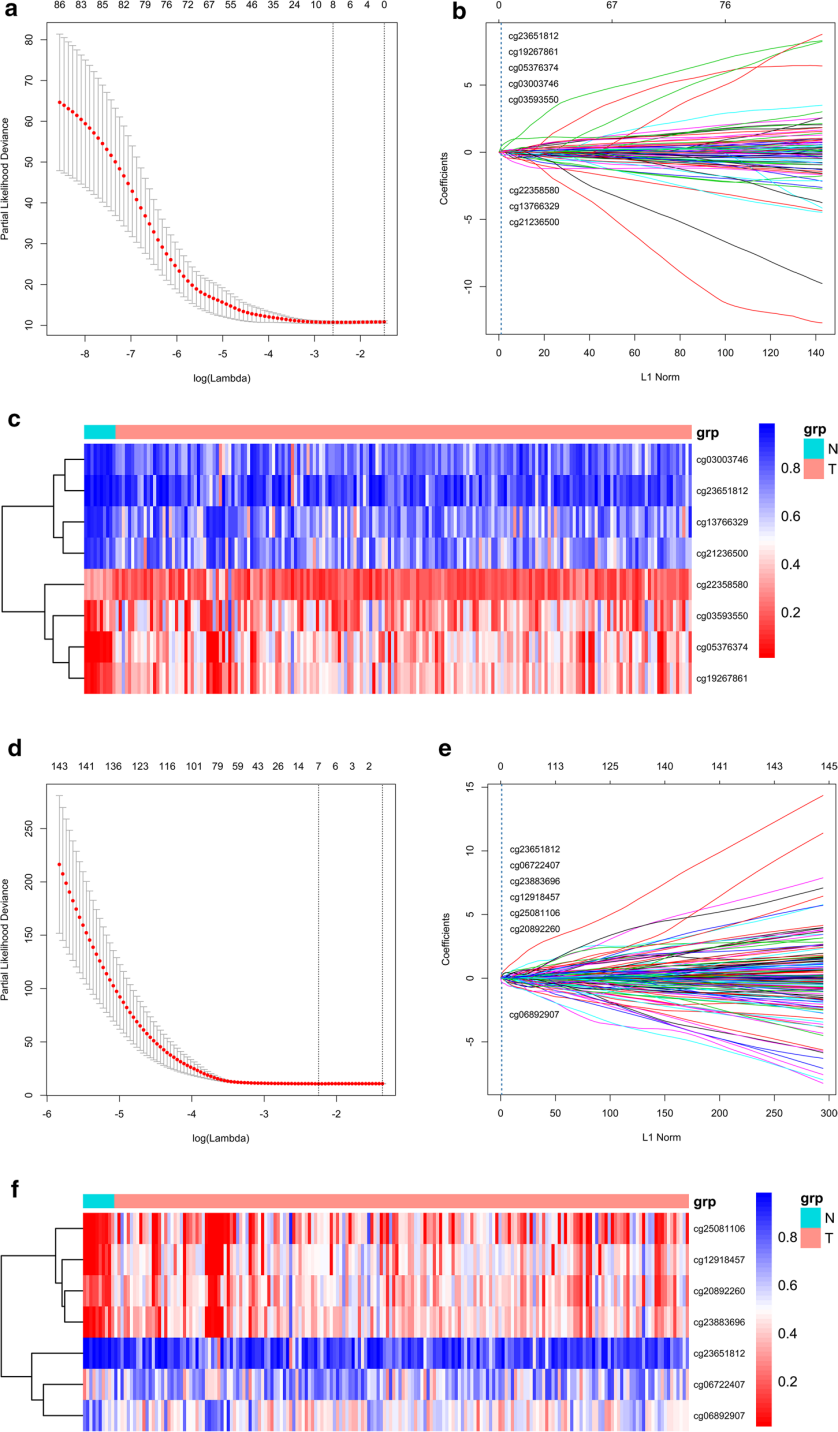
Construction of the methylation-based classifiers. **a**–**c** Methylation-based classifier of miRNA. **d**–**f** Methylation-based classifier of lncRNA. **a**, **d** Selection CpG sites in LASSO model. **b**, **e** LASSO coefficient profiles of CpG sites. **c**, **f** Hierarchical clustering using differentially methylation levels of CpG sites

To develop a methylation-based classifier to predict the prognosis of PAC, a LASSO regression model was carried out by the methylation data of 86 CpG sites of miRNA and 348 CpG sites of lncRNA, respectively. 8 CpG sites of miRNA and 7 CpG sites of lncRNA were found with regression coefficients (Fig. 1a, b, d, e). The risk score formulas for methylation-based classifiers were established as follows: methylation-based classifier for miRNA = 0.057 × beta_cg03003746 + 0.033 × beta_cg03593550 + 0.081 × beta_cg05376374 − 0.155 × beta_cg13766329 + 0.099 × beta_cg19267861 − 0.214 × beta_cg21236500 − 0.072 × beta_cg22358580 + 0.469 × beta_cg23651812, methylation-based classifier for lncRNA = 0.075 × beta_cg06722407 − 0.266 × beta_cg06892907 + 0.046 × beta_cg12918457 + 0.010 × beta_cg20892260 + 0.283 × beta_cg23651812 + 0.071 × beta_cg23883696 + 0.040 × beta_cg25081106. The characteristics of the CpG sites selected by LASSO analysis are shown in Tables 2 and 3. The methylation levels of 3 CpG sites were upregulated, and 5 CpG sites were downregulated for miRNA in PAC tissues compared to normal adjacent tissues (Additional file 1: Figure S1). For lncRNA in PAC tissues, the methylation levels of 5 CpG sites were augmented, and those of 2 CpG sites were decreased compared to normal adjacent tissues (Additional file 2: Figure S2). The methylation data of the CpG sites selected by LASSO analysis could clearly discriminate PAC and normal adjacent samples, which was further confirmed by unsupervised hierarchical clustering analyses (Fig. 1c, f).

**Table 2:** Characteristics of CpG sites for miRNA selected by LASSO

CG_ID	Gene_Symbol	CG_Chromosome_location	Position_to_TSS	CGI_Coordinate	Feature_Type
cg03003746	miR-200a	chr1: 1,167,453–1,167,454	TSS1500	chr1:1,162,663–1,165,204	S_Shelf
cg03593550	miR-935	chr19: 53,982,150–53,982,151	TSS200	chr19:53,982,049–53,982,954	Island
cg05376374	miR-129-2	chr11: 43,581,370–43,581,371	TSS200	chr11:43,580,995–43,581,665	Island
cg13766329	miR-1249	chr22: 45,201,099–45,201,100	TSS200	chr22:45,202,840–45,203,199	N_Shore
cg19267861	miR-124-3	chr20: 63,178,372–63,178,373	TSS200	chr20:63,174,902–63,179,515	Island
cg21236500	miR-4479	chr9: 136,885,445–136,885,446	TSS1500	chr9:136,885,954–136,887,075	N_Shore
cg22358580	miR-615	chr12: 54,032,941–54,032,942	TSS1500	chr12:54,033,241–54,034,925	N_Shore
cg23651812	miR-429	chr1: 1,168,986–1,168,987	TSS200	chr1:1,162,663–1,165,204	S_Shelf

*CGI* CpG island

**Table 3:** Characteristics of CpG sites for lncRNA selected by LASSO

CG_ID	Gene_Symbol	CG_Chromosome_location	Position_to_TSS	CGI_Coordinate	Feature_Type
cg06722407	LINC00421	chr13: 19,344,446–19,344,447	TSS1500	chr13:19,344,445–19,345,081	Island
cg06892907	KB-1732A1.1	chr8: 102,805,834–102,805,835	TSS1500	chr8:102,806,392–102,807,267	N_Shore
cg12918457	LINC00900	chr11: 115,760,030–115,760,031	TSS1500	chr11:115,759,680–115,760,399	Island
cg20892260	RP11-175E9.1	chr8: 23,706,512–23,706,513	TSS1500	chr8:23,704,962–23,707,662	Island
cg23651812	RP11-465B22.8	chr1: 1,168,986–1,168,987	TSS1500	chr1:1,162,663–1,165,204	S_Shelf
cg23883696	RP11-676J15.1	chr18: 72,867,063–72,867,064	TSS1500	chr18:72,866,730–72,869,636	Island
cg25081106	MIR4500HG	chr13: 87,671,914–87,671,915	TSS1500	chr13:87,671,314–87672,716	Island

*CGI* CpG island

### Predictive and prognostic value of methylation-based classifier

**Fig. 2:**
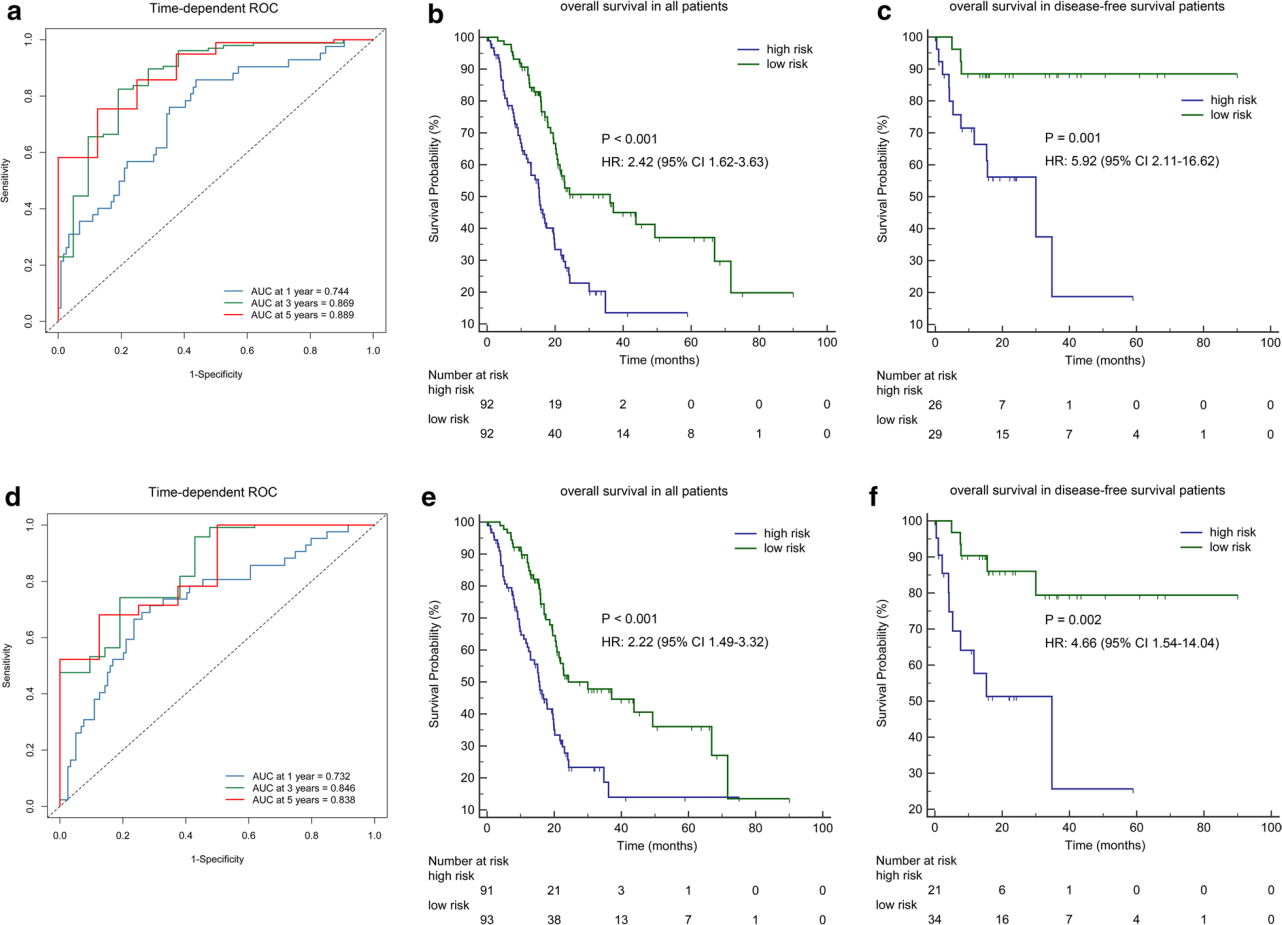
Time-dependent ROC curves and the survival analysis for the methylation-based classifiers for OS. **a**–**c** Methylation-based classifier of miRNA. **d**–**f** Methylation-based classifier of lncRNA. **a**, **d** Time-dependent ROC curves were applied to assess predictive accuracy for overall survival at varying follow-up times. **b**, **e** Kaplan–Meier analysis the overall survival in all patients. **c**, **f** Kaplan–Meier analysis the overall survival in disease-free survival patients. The median of the classifier risk score as a cut-off value to divide the patients into high-risk groups and low-risk groups

With the help of the methylation-based classifiers, the risk score of each PAC patient was calculated. And tdROC analysis was adopted to determine the accuracy of the classifiers for predicting the prognosis of PAC. The results obtained from the tdROC curve analysis indicated that these classifiers had favourable predictive and prognostic accuracy at varying follow-up times. For the methylation-based classifier of miRNA, the AUCs were 0.744 (95% CI 0.657–0.830) at 1 year, 0.869 (95% CI 0.777–0.961) at 3 years, and 0.889 (95% CI 0.784–0.995) at 5 years (Fig. 2a). For the methylation-based classifier of lncRNA, the AUCs were 0.732 (95% CI 0.641–0.823) at 1 year, 0.846 (95% CI 0.756–0.936) at 3 years and 0.838 (95% CI 0.707–0.968) at 5 years (Fig. 2d).

**Fig. 3:**
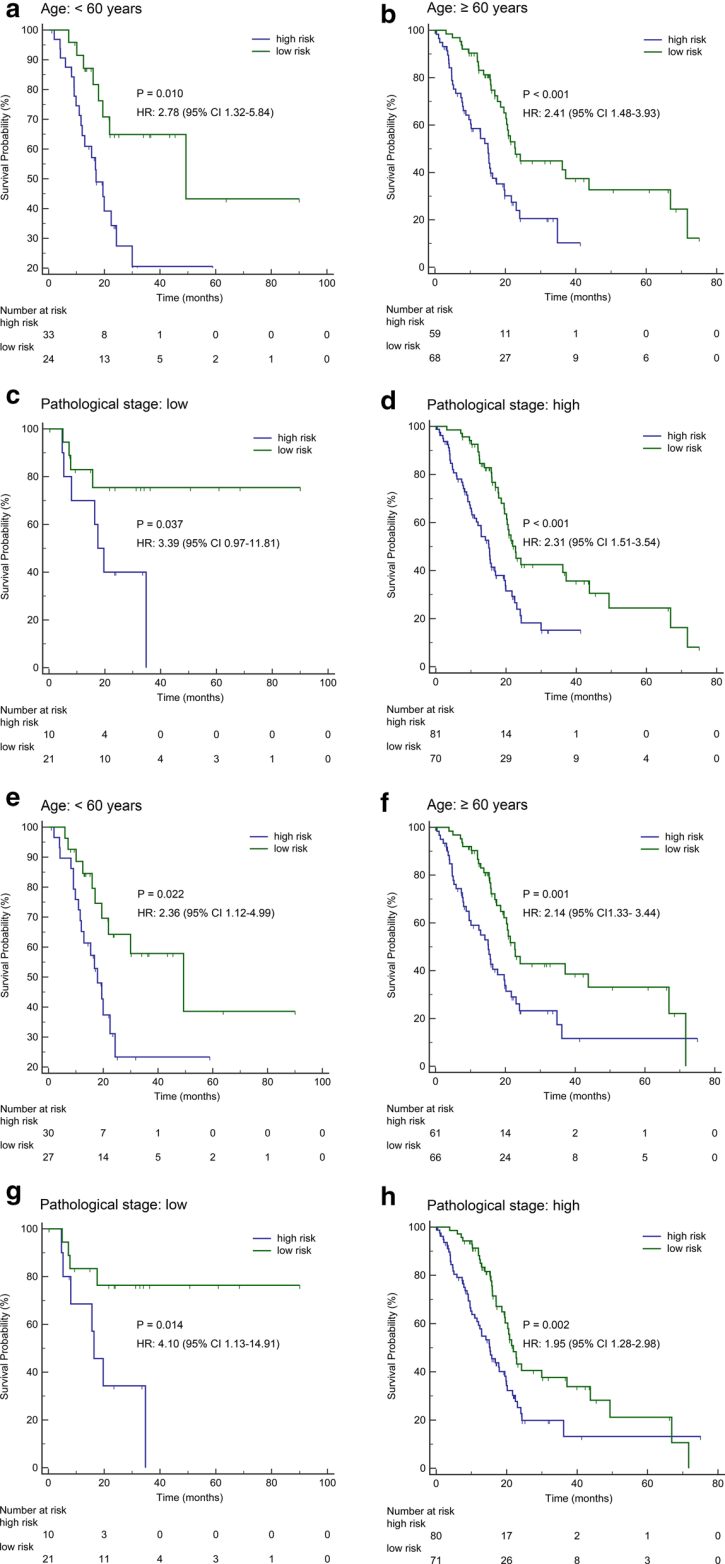
The survival analysis on the basis of the methylation-based classifiers stratified by clinicopathological risk factors. **a**–**d** Methylation-based classifier of miRNA. **e**–**h** Methylation-based classifier of lncRNA. **a**, **b**, **e**, **f** Age. **c**, **d**, **g**, **h** Pathological stage. According to the cut-off value, we calculate high and low risk of methylation-based classifier

Using the median risk score cut-off point, we categorized the PAC patients into two groups, including a high-risk group and a low-risk group. As demonstrated by Kaplan–Meier curves, patients with high-risk scores for the methylation-based classifier of miRNA had worse OS than those who had low-risk scores (HR: 2.42, 95% CI 1.62–3.63, P < 0.001, Fig. 2b). Analogously, the OS of patients with high-risk scores for the methylation-based classifier of lncRNA was shown to be worse than those who had low-risk scores (HR: 2.22, 95% CI 1.49–3.32, P < 0.001, Fig. 2e). For disease-free survival (DFS) patients, the methylation-based classifier of miRNA or lncRNA could also accurately distinguish OS according to high-risk or low-risk scores (HR: 5.92, 95% CI 2.11–16.62, P = 0.001, Fig. 2c; HR: 4.66, 95% CI 1.54–14.04, P = 0.002, Fig. 2f). To further assess the prognostic value of the methylation-based classifiers of miRNA or lncRNA, we stratified patients using clinicopathological risk factors including age and tumour size. Notably, the methylation-based classifier of miRNA (Fig. 3a–d) and LncRNA (Fig. 3e–h) still remained significant for OS.

**Fig. 4:**
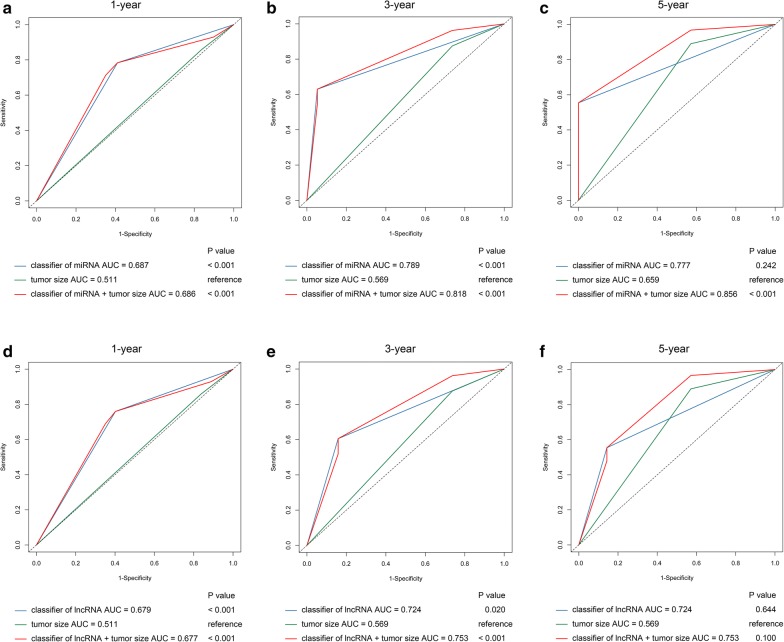
Time-dependent ROC curves compare the prognostic accuracy of the methylation-based classifiers with clinicopathological risk factors. **a**–**c** Methylation-based classifier of miRNA. **d**–**f** Methylation-based classifier of lncRNA. **a**, **d** 1-year overall survival. **b**, **e** 3-year overall survival. **c**, **f** 5-year overall survival

To compare the prognostic value of methylation-based classifiers and other clinical variables for OS, univariate Cox regression was performed. And the results revealed that tumour grade (HR: 1.56, 95% CI 1.03–2.36, P = 0.037), tumour size (HR: 2.12, 95% CI 1.13–3.98, P = 0.020), methylation-based classifier of miRNA (HR: 2.56, 95% CI 1.68–3.90, P < 0.001) and methylation-based classifier of lncRNA (HR: 2.27, 95% CI 1.51–3.40, P < 0.001) were correlated with OS. Additionally, when the clinal variables and methylation-based classifier of miRNA were adjusted by using multivariable analysis, only our methylation-based classifier of miRNA (HR: 2.78, 95% CI 1.80–4.27, P < 0.001) and tumour size (HR: 2.23, 95% CI 1.52–4.31, P = 0.017) were identified as potent and independent factors of OS (Table 4). Similarly, the results of multivariate Cox regression models presented that the methylation-based classifier of lncRNA (HR: 2.32, 95% CI 1.53–3.50, P < 0.001) and tumour size (HR: 2.07, 95% CI 1.07–4.00, P = 0.032) remained significant independent prognostic factors of OS (Table 5). And tdROC revealed that the methylation-based classifier of miRNA combined with tumour size provided increased accuracy for prediction of OS of PAC at 1 year (AUC = 0.686, 95% CI 0.600–0.772), 3 years (AUC = 0.818, 95% CI 0.736–0.899) and 5 years (AUC = 0.856, 95% CI 0.766–0.946) (Fig. 4a–c), and the methylation-based classifier of lncRNA combined with tumour size also provided a more accurate prediction for OS at 1 year (AUC = 0.677, 95% CI 0.590–0.764) and 3 years (AUC = 0.753, 95% CI 0.641–0.865) (Fig. 4d, e). We were unable to precisely predict 5-year survival rate of PAC patients using the methylation-based classifier of lncRNA combined with tumour size (Fig. 4f).

**Table 4:** Univariate and multivariate Cox regression analyses of methylation-based classifier of miRNA for overall survival

Prognostic parameter	Univariate analysis			Multivariate analysis		
	HR	95% CI	P value	HR	95% CI	P value
Age (≥ 60 vs. < 60)	1.36	0.88–2.11	0.165			
Gender (male vs. female)	0.85	0.57–1.26	0.416			
Tumor grade (G3 + G4 vs. G1 + G2)	1.56	1.03–2.36	0.037			
Tumor size (T3 + T4 vs. T1 + T2)	2.12	1.13–3.98	0.020	2.23	1.52–4.31	0.017
Pathological stage (III + IV vs. I + II)	1.00	0.40–2.46	0.991			
Classifier of miRNA (high vs. low risk)	2.56	1.68–3.90	< 0.001	2.78	1.80–4.27	< 0.001

**Table 5:** Univariate and multivariate Cox regression analyses of methylation-based classifier of lncRNA for overall survival

Prognostic parameter	Univariate analysis			Multivariate analysis		
	HR	95% CI	P value	HR	95% CI	P value
Age (≥ 60 vs. < 60)	1.36	0.88–2.11	0.165			
Gender (male vs. female)	0.85	0.57–1.26	0.416			
Tumor grade (G3 + G4 vs. G1 + G2)	1.56	1.03–2.36	0.037			
Tumor size (T3 + T4 vs. T1 + T2)	2.12	1.13–3.98	0.020	2.07	1.07–4.00	0.032
Pathological stage (III + IV vs. I + II)	1.00	0.40–2.46	0.991			
Classifier of lncRNA (high vs. low risk)	2.27	1.51–3.40	< 0.001	2.32	1.53–3.50	< 0.001

### Identification and functional evaluation of miRNA target genes and genes co-expressed with lncRNA

**Fig. 5:**
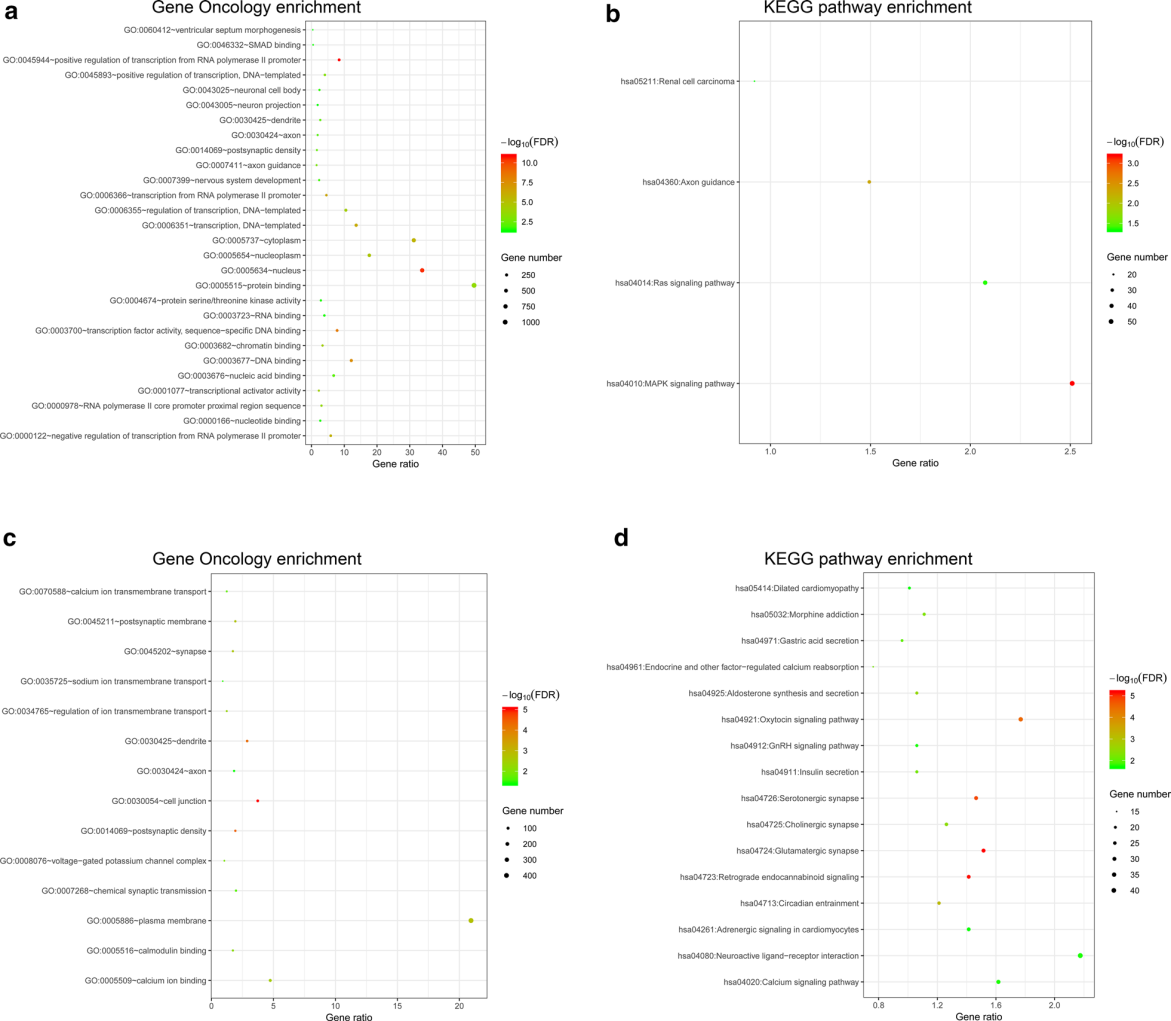
Functional enrichment analysis of miRNA target genes and lncRNA co-expression genes. **a**, **b** miRNA target genes. **c**, **d** lncRNA co-expression genes. **a**, **c** Gene Oncology (GO) enrichment. **b**, **d** KEGG pathway enrichment

There were 8 miRNAs associated with the CpG sites in the methylation-based classifier of miRNA (Table 2), and 7 lncRNAs were correlated with the CpG sites in the methylation-based classifier of lncRNA (Table 3). DIANA microT-CDS was used to identify all of the 2442 predicted miRNA target genes. MEM analysis was performed and identified a total of 2524 genes co-expressed with the lncRNAs. The functional enrichment analysis of those targets and co-expressed genes was performed by DAVID, which revealed significant enriched Gene Oncology (GO) terms and KEGG pathways. For miRNA targets, the GO biological processes were related to regulation of the nucleoplasm (cellular component), transcription (biological process), and protein serine/threonine kinase activity (molecular function), and the KEGG pathways including the MAPK signalling pathway and the Ras signalling pathway (Fig. 5a, b). For lncRNA co-expressed genes, the GO terms were related to calcium ion transmembrane transport (biological process), cell junction (cellular component), and calcium ion binding (molecular function), and the KEGG pathways including the oxytocin signalling pathway, insulin secretion and calcium signalling pathway (Fig. 5c, d).

## Discussion

PAC is one of the most invasive and lethal carcinomas worldwide, with multiple types of molecular and cellular heterogeneity [14]. Due to limited treatment strategies, the incidence of PAC is increasing yearly and most patients died within 1 year. Therefore, revealing the pathogenic mechanism of PAC will help to advance new treatment strategies. In recent decades, researches have shown that ncRNAs play crucial roles in various cancers with the great improvements in RNA-seq technologies [15]. Although several studies have focused on the functional effect of ncRNA methylation [16], the interrelation between the methylation of ncRNAs and PAC remains still elusive. DNA methylation has been proved to be critical for the regulation of protein-coding gene expressions. Accumulating evidence has illustrated that abnormal methylation at promoter CpG islands of ncRNAs may be involved in the pathogenesis and prognosis of cancers [11], and this information may contribute to prognostic prediction as well as individualized therapy in these patients.

In this study, we conducted a comprehensive and detailed analysis of the DNA methylation profile of ncRNAs in a cohort of PAC specimens from the TCGA database to investigate the altered DNA methylation patterns in PAC. Furthermore, we identified several important clinically relevant observations. Firstly, according to the 485,577 CpG sites 2 kb upstream of the TSS in ncRNAs (miRNA or lncRNA), 511 CpG sites of miRNA and 1441 CpG sites of lncRNA were filtered out using different methylation degrees between PAC and the normal neighbouring tissues. The LASSO regression method was used to further determine the ncRNA signature, which identified 8 CpG sites of miRNA and 7 CpG sites of lncRNA with regression coefficients: miR-200a, miR-935, miR-124-3, miR-129-2, miR-1249, miR-4479, miR-615, miR-429, LINC00421, KB-1732A1.1, LINC00900, RP11-175E9.1, RP11-465B22.8, RP11-676J15.1, and MIR4500HG. Reports have indicated that most of the 8 miRNAs are closely related to tumorigenesis [17–21] while none of the lncRNAs have been studied. Among the 8 miRNAs, miR-200a plays a vital part in the mesenchymal-to-epithelial transition process of pancreatic cancer stem cells by altering cancer migration and invasion [22]. Additionally, miR-935 participates in cell proliferation, migration and apoptosis by reacting with its target, INPP4A, in pancreatic cancer [18]. Emerging evidence illustrated that many ncRNAs are targets of methylation-associated silencing in several diseases, and hypermethylation of the CpG sites could lead to decreased expression of ncRNA [23]. In our study, we confirmed that the methylation levels of CpG sites were negatively corrected with the expression of miRNA or lncRNA when we screened out the CpG sites of miRNAs and lncRNAs, and this outcome is consistent with other studies. It is reported that promoter hypermethylation of miR-766-3p downregulated the expression of miR-766-3p, indicating the poor prognosis in renal cell carcinoma [24]. Therefore, we speculated that these two methylation-based classifiers of ncRNA can be used to determine the prognosis of pancreatic cancer, possibly by altering the expression of ncRNA to indirectly affect the progression of pancreatic cancer. In addition, the expression pattern of the typical ncRNAs, which were correlated with the CpG sites in the methylation-based classifiers may also could be used for the prognosis of PAC patients. Secondly, the developed methylation-based classifiers of the 8 miRNAs or the 7 lncRNAs can predict the prognosis of PAC precisely, which was the highlight of this study. And tdROC analysis also confirmed the predictive accuracy of the classifiers for OS in PAC patients and in PAC patients with DFS, which is conducive to guiding the implementation of treatment strategies at different stages of the disease. Additionally, the patients can be divided into two groups by this new method based on the median risk score cut-off point. We found that the OS of the patients in the low-risk groups outperformed that of the high-risk group. Consistently, the classifiers could also distinguish difference in OS between the two risk groups according to the clinicopathological risk factors based on the Kaplan–Meier curves, which could help to determine credible individual measures for the patients. Notably, it is urgent to develope additional treatments to improve preoperative risk assessment for PAC patients in low-risk groups. Meanwhile, the subgroup of PAC patients with high-risk scores identified with these two classifiers might be candidates for more aggressive treatment strategies. There is absolutely no doubt that our miRNA and lncRNA classifiers possessed their own unique prediction compared with the clinicopathological risk factor. And when the two classifiers combination with clinicopathological risk factors, it would provide a more accurate prediction for OS at different times for PAC patients. Surprisingly, we found that the methylation-based classifier of miRNA exhibited better predictive power than that of lncRNA, which is partially due to the indirect effect of lncRNA. Because lncRNA could interact with miRNA as a competitive endogenous RNA and affect mRNA expression [25]. Therefore, the two methylation-based classifier signatures have shown a favourable effect on survival prediction, which will contribute to therapeutic decision-making.

Tumorigenesis is a complex biological process including multifarious epigenetic alterations. Methylation of miRNAs and lncRNAs may participate in the development of carcinoma and play a crucial role in the disease progression. To evaluate the biological function of the ncRNAs in PAC, DAVID performed functional enrichment analysis of the target genes and co-expression genes. And 2442 target genes and 2524 co-expressed genes with abnormal methylation of miRNA and lncRNA, respectively, were identified. The GO results showed the major differences in tumour biology in terms of cellular component, biological process and molecular function. Based on the KEGG pathway analysis results, insulin secretion and the MAPK, Ras, and calcium signalling pathways were significantly enriched, which have previously been validated as vital to the tumorigenesis of PAC [26–29]. Notably, the MAPK and Ras signalling pathways are frequently affected in PAC. Yang et al. [30] reported that, in PAC, the tumour suppressor RKIP is regulated by the Ras coded protein, named KRAS, probably via the MAPK-ERK signalling pathway. Moreover, several miRNAs were demonstrated to function as regulators of oncogenic KRAS signalling in PAC [27], which provides novel therapeutic evidence for targeting the Ras signalling pathway in patients. Therefore, these two candidate prognostic classifiers will be beneficial for improving prognosis prediction accuracy and guiding individualized treatment in PAC.

However, there are several limitations in this study. Although we identified aberrantly methylated sites of miRNAs and lncRNAs, whether the alterations represent a cause or an outcome of the cancer process remains unknown. Actually, methylation can occur in both the intergenic regions and repetitive sequences of the human genome and gene promoters. And aberrant hypermethylation of CpG sites in gene promoters may silence the gene expression that is critical to cell homeostasis, DNA integrity, or genome stability [31]. On the other hand, hypomethylation of CpG sites in the intergenic regions and repetitive sequences may lead to genomic instability and the loss of gene imprinting [32]. Furthermore, gene expression is related to the degree of methylation [33], which may be discrepant in different genes. Regrettably, this study mainly focused on the association between the methylation level of ncRNA and the prognosis of pancreatic cancer. It will be interesting to study the correlation of these different methylation styles in the future. In addition, no additional experimental data analysis on the underlying mechanisms of miRNAs and lncRNAs was performed in this study. It is urgent to conduct further experimental studies to address these issues.

## Conclusion

In summary, our study identified two methylation-based classifiers of ncRNA associated with OS in PAC. Notably, the altered DNA methylation patterns could accurately predict the prognosis of PAC patients. These patterns are also involved in major cancer signalling pathways known to be crucial in carcinogenesis. Although more experimental studies are required for further confirmation, this is the first study to examine the relationship between aberrant DNA methylation of ncRNAs and the prognosis of PAC patients, and the results may contribute to the development of individual therapy.

## Additional files

**Additional file 1: Figure S1.** The difference beta-values of the 8 CpG sites of methylation-based classifier of miRNA between pancreatic adenocarcinoma tissues and normal adjacent tissues.

**Additional file 2: Figure S2.** The difference beta-values of the 7 CpG sites of methylation-based classifier of lncRNA between pancreatic adenocarcinoma tissues and normal adjacent tissues.

## Abbreviations

ncRNAs: non-coding RNAs
PAC: pancreatic adenocarcinoma
OS: overall survival
miRNAs: microRNAs
lncRNAs: long non-coding RNAs
GCA: gastric cardia adenocarcinoma
TCGA: The Cancer Genome Atlas
LASSO: Least Absolute Shrinkage and Selection Operator
TSS: transcriptional start site
ROC: receiver operating characteristic
AUC: the area under the curve
MEM: Multi-Experiment Matrix
FDRs: false discovery rates
DFS: disease-free survival
GO: Gene Oncology
