# Supplementary figures and images for: The DNA methylation profile of non-coding RNAs improves prognosis prediction for pancreatic adenocarcinoma
Source: Cancer Cell Int. 2019 Apr 23;19:107. doi: 10.1186/s12935-019-0828-8 (PMC6480888; doi:10.1186/s12935-019-0828-8)

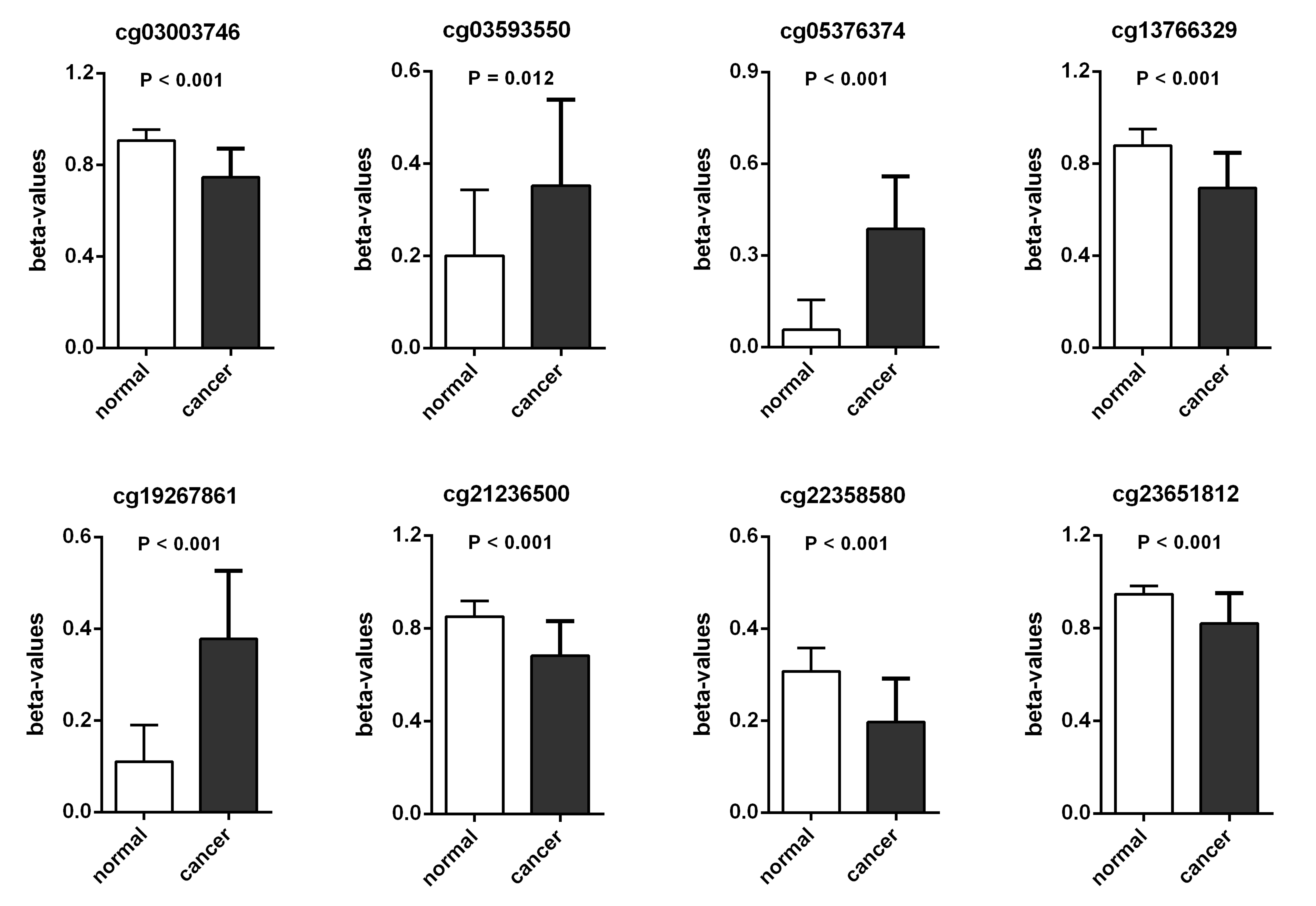

Supplement: Supplementary file 1 — Additional file 1: Figure S1. The difference beta-values of the 8 CpG sites of methylation-based classifier of miRNA between pancreatic adenocarcinoma tissues and normal adjacent tissues. [file 12935_2019_828_MOESM1_ESM.tif]

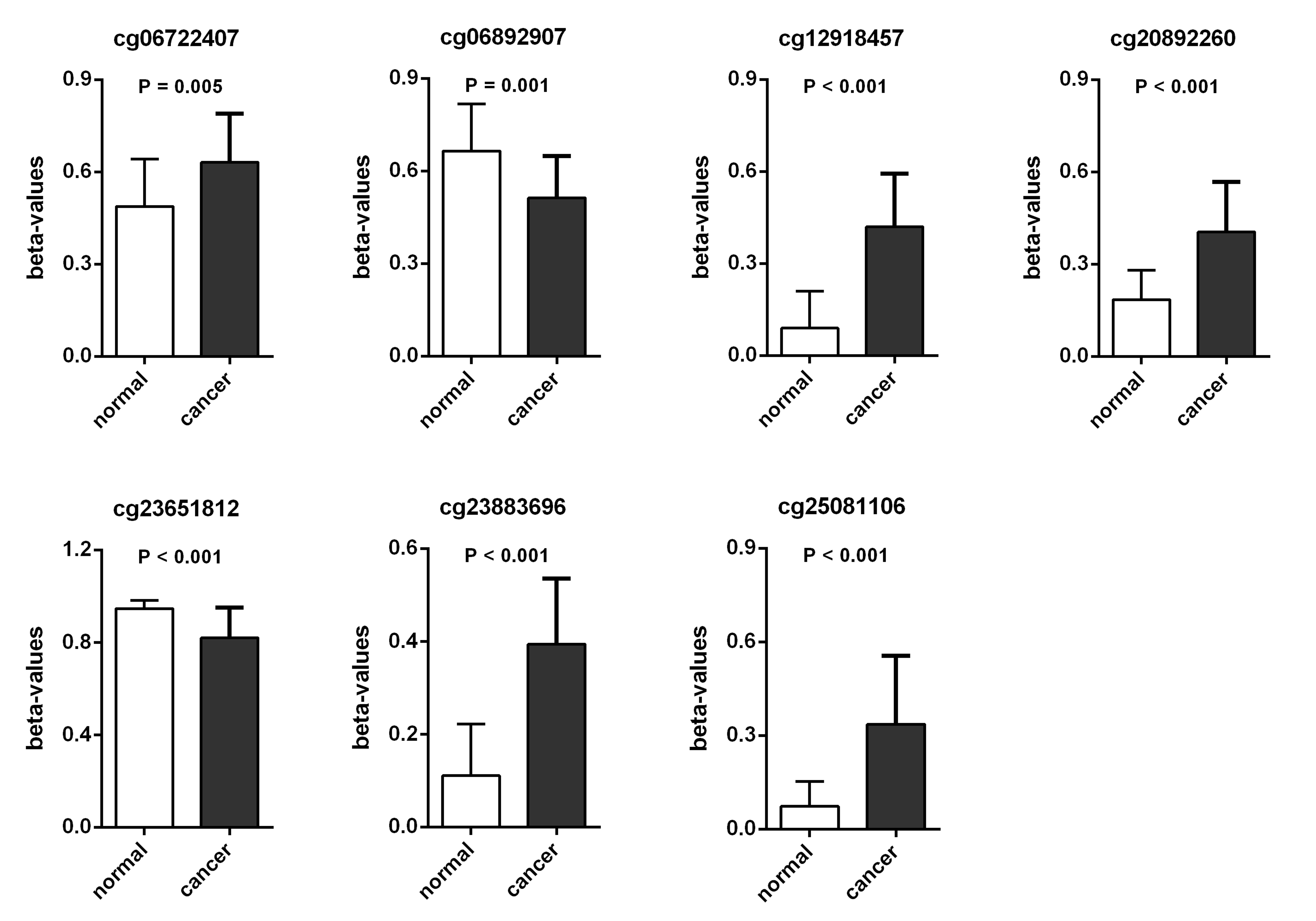

Supplement: Supplementary file 2 — Additional file 2: Figure S2. The difference beta-values of the 7 CpG sites of methylation-based classifier of lncRNA between pancreatic adenocarcinoma tissues and normal adjacent tissues. [file 12935_2019_828_MOESM2_ESM.tif]
